# Cross-Generational Validation of a Feedforward Neural Network for Milk Yield Prediction in Dairy Cattle

**DOI:** 10.3390/ani16050707

**Published:** 2026-02-25

**Authors:** Carlotta Ferrari, Chiara Punturiero, Andrea Delledonne, Andrea Mario Vergani, Marco Masseroli, Maria G. Strillacci, Alessandro Bagnato

**Affiliations:** 1 Department of Veterinary Medicine and Animal Sciences, University of Milan, 26900 Lodi, Italy; chiara.punturiero@unimi.it (C.P.); andrea.delledonne@unimi.it (A.D.); alessandro.bagnato@unimi.it (A.B.); 2 Health Data Science Centre, Human Technopole, Viale Rita Levi-Montalcini 1, 20157 Milan, Italy; andreamario.vergani@mail.polimi.it; 3 Department of Electronics, Information and Bioengineering, Politecnico di Milano, 20133 Milan, Italy; marco.masseroli@polimi.it

**Keywords:** milk prediction, feedforward neural network, precision livestock farming

## Abstract

Advancements in precision livestock farming and machine learning have expanded the use of data-driven approaches for milk yield forecasting. In this study, a previously developed feedforward neural network (FFNN) model using genomic breeding values, parity, days in milk, month of calving, and age at calving as predictors was validated across one generation of Holstein cows. Specifically, the model was evaluated in first-parity daughters of the animals included in the original training population. Predictive performance was assessed on 228 lactation curves comprising 67,010 daily observations using a train–cross-validation–held-out test framework. On the test set, the model achieved a daily root mean squared error (RMSE) of 5.98 kg/day, with a Pearson correlation of 0.64. Sensitivity analyses were conducted by systematically shifting calving month and age (±1 to ±4 months) while holding other predictors constant. Simulated scenarios suggested increased predicted milk yield with later calving ages; however, these results reflect the structure of the training data rather than prescriptive management recommendations. While the FFNN provides robust milk yield predictions, its practical application for calving strategy decisions should be integrated with economic and reproductive considerations. Overall, the findings support the generational robustness of FFNN-based milk yield forecasting within the studied herd.

## Simple Summary

Accurately predicting milk production helps dairy farmers improve herd management and efficiency. In this study, we tested a previously developed machine learning model that predicts daily milk yield using genetic information and production data automatically recorded during milking. The model was applied to a new generation of cows, specifically the daughters of the animals used to develop the original model, raised under the same farm conditions. The predictions were accurate and closely matched those obtained in the original study, showing that the model remains reliable across generations. We also explored how predicted milk yield changes when the age and season of calving are modified to better understand the model’s behavior. These findings suggest that data-driven prediction tools can support dairy management decisions, as long as they are used together with economic and reproductive considerations.

## 1. Introduction

Precision livestock farming and machine learning (ML) have revolutionized milk production forecasting, enabling improved herd management, economic planning, and resource optimization [1]. Over the years, various parametric models have been developed to describe lactation curves at both the herd and individual levels. Notable contributions include the lactation models proposed by Wood, Ali & Schaeffer, and Wilmink, which have been extensively validated and widely used in lactation studies [2,3,4]. More recently, lactation curve modeling has evolved through the use of high-frequency test-day data, flexible longitudinal frameworks, and computationally efficient mixed effects implementations, enabling improved modeling of each cow’s heterogeneity and deviations from average lactation trajectories. In particular, random regression and spline-based approaches have been increasingly applied to test-day milk yield to capture time-varying variance structures and non-linear lactation dynamics under commercial conditions [5,6,7].

The integration of Automated Milking Systems (AMSs) and digital data platforms has further enhanced predictive models, enabling continuous monitoring of key phenotypic variables such as milk yield, feed intake, and temperature. These systems generate large datasets that, when combined with parametric and ML approaches, contribute to more accurate predictions and informed decision-making in dairy herd management [8,9,10].

Machine learning methods are particularly suited to livestock applications because they can handle large, complex, noisy, and non-linear datasets generated by automated recording systems [8,9]. In this context, approaches can be broadly grouped into supervised methods, which learn from labeled outcomes to predict values or classify events, and unsupervised methods, which explore data structure to detect latent patterns and heterogeneity without predefined labels [11,12]. Both approaches are relevant in dairy production, as prediction tasks (e.g., milk yield forecasting) and exploratory analyses (e.g., identifying subgroups with distinct production or health profiles) can support decision-making within precision livestock farming frameworks.

While traditional studies focused on empirical lactation curve modeling, recent research has increasingly leveraged machine learning (ML) to integrate genetic, environmental, and phenotypic data for milk-yield prediction [13,14]. Several studies have demonstrated the advantages of ML approaches, including applications to lactation curve modeling, production efficiency, and spectral trait prediction [15,16,17,18]. In this context, feedforward neural networks (FFNNs) are data-driven models capable of learning complex non-linear relationships through fully connected layers [19,20]. Although FFNNs are widely used in other domains, their application to dairy production remains comparatively limited, particularly in studies that assess model robustness beyond the training population or explore model behaviour under structured perturbations of biologically meaningful covariates [15,21,22].

In this context, Vergani et al. [18] developed an FFNN using genomic breeding values, parity, days in milk, month of calving, and age at calving, achieving competitive prediction accuracy in a single herd. These predictors are biologically and economically relevant: age at first calving influences productivity and lifetime efficiency, with deviations from the recommended 22–26 months associated with reduced yield and higher rearing costs, while month of calving reflects environmental and management conditions shaping lactation dynamics [23,24].

Despite the increasing use of machine learning approaches in dairy systems, the extent to which these models generalize across biological generations remains unclear. Most existing studies evaluate predictive accuracy within the same population used for model development, leaving open the question of cross-generational robustness in evolving livestock populations. Furthermore, limited attention has been given to the internal consistency of neural network predictions under structured perturbations of biologically meaningful covariates.

The aim of the present study is, therefore, twofold: (i) to evaluate the generational robustness of a previously developed FFNN by validating its predictive performance in first-parity daughters of the original training animals and (ii) to conduct a structured sensitivity analysis by systematically shifting calving age and month in order to assess whether the model captures coherent biological interactions among genetic merit, calving attributes, and lactation stage.

## 2. Materials and Methods

### 2.1. Study Workflow

In this study, we validated the FFNN model for daily milk yield prediction proposed by Vergani et al. [25], using a new generation of primiparous cows. To do so, we re-trained the original prediction model on 400 first lactations from the original dataset by Vergani et al. [25] and later tested the FFNN on 228 lactation curves from the daughters of the animals on which the model was trained. The FFNN predicted daily milk yield from genetic information and production data recorded during milking. Training and testing cows were raised under the same farm conditions.

In addition to testing the predictive performance of the FFNN on a new generation of cows, we also leveraged the model to run simulations for understanding how predicted lactation curves changed with varying age and season of calving.

Summarizing, the study was developed following these steps:

1. FFNN training on 400 first lactations, following the approach proposed by Vergani et al. [25] (see Section 2.2 “Machine learning model”).
2. Selection of a test set for model validation, comprising 228 first lactations from a new generation of cows (i.e., daughters of the animals considered for training the FFNN) (see Section 2.3 “Test set”).
3. FFNN validation on the test lactations, in terms of daily and cumulative—on 305 days—milk yield predictions (see the first part of Section 2.4 “Inference and simulation” and Section 2.5 “Model Validation and Performance Assessment”).
4. Simulation of new lactation curves from the FFNN, in the case of shifting calving date in the range of [−4; +4] months (see the second part of Section 2.4 “Inference and simulation”).

### 2.2. Machine Learning Model

The prediction model referred to in this work—a FFNN with one hidden layer—was proposed by Vergani et al. [25] to predict the daily milk yield of a bovine given gEBV for milk yield at 305 days, parity, DIM, age at calving (in months), and month of calving. The daily milk production data used for the original study were collected between February 2020 and March 2022 from a single Holstein Friesian herd (502 cows) in the Lombardy region (Italy), equipped with AMS. Keeping the same model configuration (i.e., predictors, target, training procedure, and hyperparameter tuning) as Vergani et al. [25], except for excluding parity from the set of covariates, we re-trained the FFNN only on the first 400 lactations coming from the original dataset. We chose to re-train the model only on data from primiparous bovines to better capture patterns specific to first lactation milk production curves, constituting the scope of our validation and simulation analyses.

A FFNN is a machine learning model that learns a (potentially non-linear and arbitrarily complex) function *f* mapping a set of input features to an output variable using a training dataset. In the present study, the model predicts daily milk yield as a function of genomic breeding value for milk yield at 305 days (gEBV), days in milk (DIMs), age at calving, and month of calving:

daily_milk_yield=f(gEBV,DIM,age_at_calving,month_of_calving)

The network parameters are estimated by minimizing the mean squared error between predicted and observed daily milk yield in the training set. Once trained, the learned function f can be applied to new observations to generate daily milk yield predictions. We focused on the FFNN architecture for milk yield prediction because Vergani et al. [25] already demonstrated its superior performance compared to other machine learning (i.e., random forest, AdaBoost, support vector regression, K-nearest neighbors) and linear regression models, in terms of the RMSE, MAE, Pearson, and concordance correlation coefficients. Full details about the comparison between FFNNs and other models for the considered task and training dataset were reported by Vergani et al. [25].

The FFNN was developed with Python 3.9.7, mainly relying on the Keras library for model implementation, training, and evaluation, on Scikit-learn for feature preprocessing and validation, and on Pandas for data management and manipulation.

### 2.3. Test Set

Test lactation records were collected from the same Holstein Friesian dairy farm from which the training data came. Test animals were selected among the daughters of cows whose lactation records were used to train the model. Importantly, no milk yield data from these test animals were included in the training phase, ensuring separation of phenotypes between training and test sets. The test set included 81,464 total daily records across 251 lactations, recorded between 29 November 2021 and 26 February 2024. To refine the test data, only lactations that extended to at least 270 DIMs were retained, and daily records were considered up to the 305th DIM. This filtering resulted in a final test set of 228 lactation curves and 67,010 observations. Descriptive statistics of the dataset used for testing are presented in Table 1.

### 2.4. Inference and Simulation

We applied the trained neural network model to predict the daily milk yield of test animals, starting from the covariates collected (i.e., DIM, age at calving, month of calving) and the gEBV by the breeders’ association ANAFIBJ. The daily predictions were compared with the real lactation curves to evaluate the model’s predictive performance: we analyzed the daily forecasting performance and the cumulative one on 305 days, in terms of the root mean squared error (RMSE), mean absolute error (MAE), percentage MAE, and Pearson correlation. Using the neural network, we also simulated new lactation curves in the case of shifting the calving date from −4 to +4 months. Specifically, the simulation consisted of the model’s prediction with shifted age at calving and month of calving (by −4, −3, −2, −1, +1, +2, +3, or +4 months), keeping the bovine’s gEBV and DIM unchanged. The simulations were compared with the real curves to understand the potential impact of breeders’ decisions on the milk production of individual animals over full lactation.

### 2.5. Model Validation and Performance Assessment

The model’s performance was evaluated on the test set, which included 228 lactation curves. The RMSE, MAE, percentage MAE, and Pearson correlation were computed for each lactation curve, and the summary results were reported based on the daily milk yield and the cumulative milk production over 305 d.

Specifically, for the FFNN, we tuned the number of neurons in the hidden layer in a 10-fold cross-validation setting (splitting on animals) by minimizing the mean squared error and later re-trained the model with the best-performing value of this hyperparameter on the full training set. This trained FFNN was then evaluated on our held-out test set of 228 lactation curves in terms of the RMSE, MAE, percentage MAE, and Pearson correlation, both on daily and cumulative milk production. Thus, we followed a train–cross-validation–test approach for model development and evaluation, relying on the Scikit-learn Python library for cross-validation and on the Keras and Scipy Python libraries for metrics calculation.

## 3. Results and Discussions

### 3.1. Forecasting Performance

**Figure 1:**
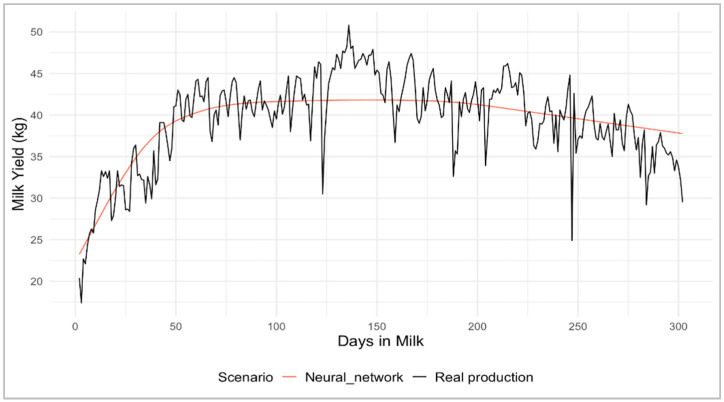
Example of a real lactation curve and its prediction of the neural network model on the test set.

To visualize an overview example of the lactation curves, daily milk yield production and predictions for one lactation curve are plotted in Figure 1, while the performance metrics are summarized in Table 2.

Table 2 shows both the model’s performance and the number of observations present in the test set. The model achieved a MAE of 4.80 kg/day, reflecting the average deviation between predicted and observed daily yields. The test cohort of first-parity daughters had an average daily milk production of 37.48 ± 7.77 kg, whereas the corresponding mothers in the original training population averaged 39.7 ± 9.1 kg. These comparable production levels suggest broadly similar yield distributions between generations, supporting the suitability of the daughter cohort for evaluating the model’s predictive consistency.

Comparable cumulative prediction accuracy has been reported in previous studies, where they used an artificial neural network with similar biological covariates. For example, Gorgulu [20] applied a feedforward artificial neural network including age, parity, season of calving, and early test-day milk yield records to predict cumulative 305-day milk yield in Brown Swiss cattle, reporting a RMSE of 1162.7 kg for the best-performing model. In the present study, the corresponding RMSE for cumulative 305-day milk yield was 1260.50 kg, a comparable magnitude given differences in breed, data structure, and validation strategy. The similarity in cumulative prediction error supports the consistency of ANN performance across studies and reinforces the suitability of the proposed FFNN framework for modeling lactation-level production outcomes.

Liseune [26] reported a Pearson correlation of 0.87 in the training set for parity 1 when using a traditional multilayer perceptron model and further improved performance through a sequential autoencoder framework designed to reconstruct and predict daily milk yield trajectories. Their sequential autoencoder achieved correlations up to 0.83–0.84 for short-term forecasts (30 days ahead) and demonstrated robust performance across varying data availability windows. In our study, the Pearson correlation for cumulative 305-day yield prediction on the independent test set was 0.70, which is lower than the performance reported by Liseune et al. but remains consistent with expectations, given that our estimate reflects true out-of-sample generalization rather than in-sample fit.

Recent work on the national scale has also shown that deep learning architectures, particularly CNN-based models, can outperform traditional autoregressive approaches for milk production forecasting [27].

This performance is also comparable to the value reported by Vergani et al. [10], who obtained an MAE of 4.42 kg/day [25]. The difference of 0.38 kg/day is modest and indicates that the FFNN retained a predictive accuracy similar to that observed in the original study, despite being applied to a new cohort of first-parity daughters.

In the original work, performance statistics for primiparous cows alone were not reported, limiting direct parity-specific comparisons. However, Vergani et al. [10] documented an RMSE of 6.47 kg/day and a Pearson correlation of 0.71 when combining first and second parities [25]. For context, the RMSE and correlation observed here are consistent with those documented in the original study.

To explore whether performance varied across biologically or economically relevant groups, metrics were further aggregated by month of calving (Table 3), age at calving (Table 4), and gEBV class (Table 5).

As expected, given the high variability of daily milk yield trajectories, standard deviations within classes were large, and no clear linear trends emerged across the evaluated categories. Similar dispersion of prediction errors across lactation stages and production levels has been documented in artificial neural network applications to dairy data, where residual variability remains high, even under well-fitted models [14].

The most represented calving age group (23–25 months), corresponding to the range commonly associated with favorable reproductive and productive outcomes, showed relatively consistent MAE (4.07–4.91 kg/day) and RMSE values (5.28–6.02 kg/day). This behavior is consistent with previous observations that neural network performance tends to be more stable in highly represented regions of the feature space, where the training data provide adequate coverage [28].

In contrast, sparsely represented categories, such as extreme calving ages, exhibited greater variability in prediction error. Comparable heterogeneity in predictive performance across biological strata has been observed in previous dairy applications of neural networks, where error magnitude varies across lactation stages, parities, or data availability conditions [14,26,29].

Similar considerations apply to the gEBV stratification. While no systematic pattern across genetic merit classes was detected, the most populated gEBV range (904–1304 kg) exhibited moderate and relatively homogeneous MAE (4.36–4.96 kg/day) and RMSE values (5.58–6.15 kg/day), whereas sparsely represented high-gEBV classes (>1504 kg) showed higher prediction errors. Previous ANN-based studies have reported variability in prediction accuracy across production levels and data structures, suggesting that differences in sample representation may influence error dispersion [20,28]. In this context, the increased variability observed in extreme gEBV classes is more plausibly attributable to differences in data density than to systematic biological non-linear responses.

### 3.2. Simulation of the Impact of Shifting the Month and Age at Calving

**Figure 2:**
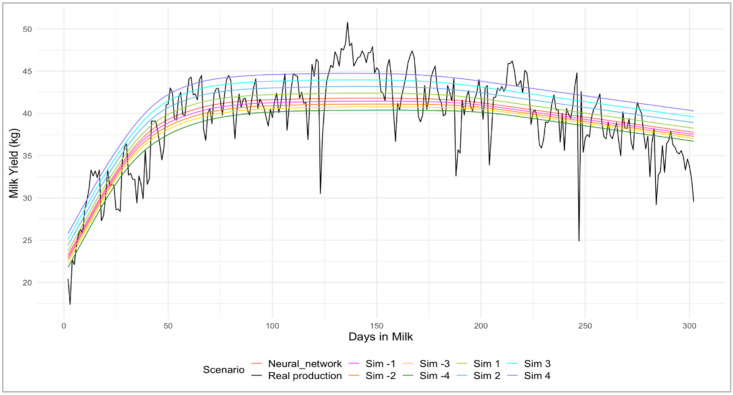
Example of a cumulative milk production recorded and predicted using neural networks, with varying calving month shifts in the simulation (sim.).

Shifting the month and age at calving by −4 to +4 months allowed us to evaluate the response of the FFNN to systematic perturbations of these covariates while keeping gEBV and DIM constant. These simulations were designed as sensitivity analyses to explore the internal behavior of the neural network under controlled covariate shifts rather than as prescriptive management scenarios. An example of simulation is shown in Figure 2.

**Figure 3:**
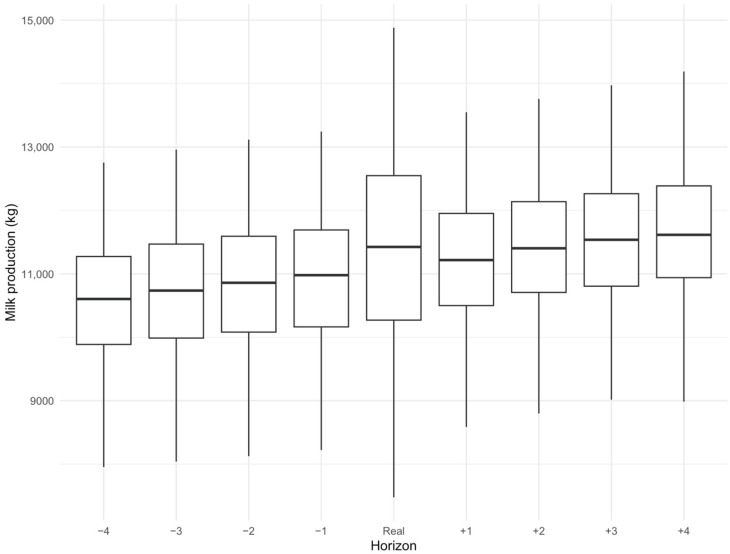
Distribution of cumulative milk production grouped by the simulated horizon. Simulated horizons are the months of calving and consequently the age at calving shifted by −4, −3, −2, −1, +1, +2, +3, or +4 months.

Across all simulated horizons, predicted cumulative 305-day yield showed a consistent dependence on age at calving, with higher simulated ages associated with increased predicted production as shown in Figure 3. This trend aligns with the established dairy literature demonstrating that milk yield in first lactation is strongly influenced by age at first calving [23,30]. However, although moderate delays in calving age may be associated with increased first-lactation yield, economic analyses consistently indicate that optimal profitability is achieved when first calving occurs between approximately 22 and 25 months [23,31]. Delaying calving beyond this range increases rearing costs and prolongs the non-productive period of the heifer, reducing economic efficiency.

Notably, the variability of simulated cumulative yields was narrower than that observed in the real data. Similar smoothing behavior has been reported in neural network-based modeling of lactation curves, where reconstructed trajectories tend to exhibit reduced dispersion relative to raw observations [26,28].

The model behaviour observed here likely reflects the structure of the training data. Most recorded lactations corresponded to calving ages between 23 and 25 months, whereas extreme ages were sparsely represented. Consequently, predictions generated for older or younger simulated ages relied on limited training support, which may have contributed to reduced variability and increased sensitivity in these marginal strata.

### 3.3. Real-World Applications and Computational Cost

Training and cross-validating the FFNN (including best hyperparameter selection) took a few hours on a 64-bit laptop equipped with an Intel Core i7-8650U processor and 16 GB RAM. Applying the trained model to a new lactation to obtain the predicted milk yield curve, instead, runs in the range of seconds on modern laptops. Hence, the computational cost of using our tool is limited, considering that breeders are expected to train the FFNN on their data only once and then use it to predict new lactation curves. Clearly, in order for individual farmers to easily use the proposed method in practice, our tool should be integrated into farm management software that would automatically manage inputs, train the model, and apply it to suggest optimal decisions with null or minimal manual effort to set data and parameters (and without requiring programming knowledge). Thus, we envision faster real-world applications of tools such as the one we proposed and validated in farms already technologically equipped with AMS and management software.

A limitation of the present study is that only a single modeling framework (FFNN) was evaluated. Although previous benchmarking identified this architecture as suitable for the considered task, direct comparison with alternative machine learning models under the current generational validation design was not performed. Future research should assess whether similar cross-generational robustness can be observed across different modeling approaches.

## 4. Conclusions

The FFNN model developed by Vergani et al. [25] maintained stable predictive performance when applied to first-parity daughters of the training animals, supporting its generational robustness under comparable management conditions. Prediction accuracy remained broadly consistent across calving months, calving ages, and gEBV classes, although increased variability in underrepresented groups highlights the importance of balanced training data for reliable forecasting.

Sensitivity analyses based on systematic shifts in calving age and month revealed coherent internal model behaviour. However, these simulated outcomes primarily reflect the structure of the training data and should not be interpreted as direct management recommendations. Because the model predicts milk yield alone, any practical application for decision-making should be integrated with economic and reproductive considerations.

We believe that our validation of the model across generations of cows emphasizes that novel methods for milk yield forecasting, such as FFNNs, hold potential for real-world breeders’ decision support and wider-scale precision farming in the future. Nevertheless, broader multi-herd validation and complementary analyses will be necessary before the approach can inform calving strategy decisions.

## Figures and Tables

**Table 1:** Descriptive statistics of the test set used.

Variable	Mean	SD	Min	Max
Daily milk yield (kg/day)	37.48	7.77	32.80	60.70
Cumulative production (kg/305 d)	11,763	1644	7715	14,897
Age at calving (months)	25	2	19	32
gEBV (kg)	833.3	502.8	−296.0	2132.0

**Table 2:** Performance of the neural network model on the test set. RMSE = root mean squared error for daily (kg/day) and cumulative lactation (kg/305 d); MAE = mean absolute error for daily (kg/day) and cumulative lactation (kg/305 d); MAE % = percentage of mean absolute error; Pcorr = Pearson correlation; n_obs = number of observations.

	RMSE	MAE	MAE %	Pcorr	n_obs
Daily	5.98 kg/day	4.80 kg/day	12.5	0.64	67,010
Cumulative	1260.50 kg/305 d	1029.70 kg/305 d	9.38	0.70	228

**Table 3:** Performance of the neural network model on the test set, aggregated by month of calving. RMSE = root mean squared error; MAE = mean absolute error; Pcorr = Pearson correlation; n. obs = number of observations; n. animals = number of animals in each category. RMSE and MAE are expressed as mean ± standard deviation.

Month of Calving	RMSE (kg/day)	MAE (kg/day)	Pcorr	n. Obs	n. Animals	Mean Production (kg/day)
1 (January)	6.16 ± 3.73	4.91 ± 3.73	0.56	4087	14	37.60
2 (February)	7.05 ± 3.95	5.85 ± 3.95	0.42	3499	12	38.44
3 (March)	6.73 ± 3.49	5.75 ± 3.49	0.73	1158	4	41.55
4 (April)	5.67 ± 3.14	4.72 ± 3.14	0.52	1165	4	41.85
5 (May)	6.17 ± 3.49	5.09 ± 3.49	0.42	3604	12	39.02
6 (June)	6.25 ± 3.59	5.12 ± 3.59	0.61	7746	26	41.47
7 (July)	5.86 ± 3.56	4.66 ± 3.56	0.73	7345	25	39.92
8 (August)	5.37 ± 3.36	4.19 ± 3.36	0.72	14,436	49	37.39
9 (September)	6.09 ± 3.46	5.01 ± 3.46	0.61	10,584	36	37.70
10 (October)	6.09 ± 3.42	5.04 ± 3.42	0.72	5509	19	37.41
11 (November)	6.44 ± 4.08	4.99 ± 4.08	0.58	2046	7	38.80
12 (December)	5.68 ± 3.68	4.33 ± 3.68	0.60	5831	20	35.61

**Table 4:** Performance of the neural network model on the test set, aggregated by age at calving (in months). RMSE = root mean squared error; MAE = mean absolute error; Pcorr = Pearson correlation; n. obs = number of observations; n. animals = number of animals in each category. RMSE and MAE are expressed as mean ± standard deviation; - = no data available for that class.

Age at Calving	RMSE (kg/day)	MAE (kg/day)	Pcorr	n. Obs	n. Animals	Mean Production (kg/day)
19	5.41 ± 3.12	4.42 ± 3.12	0.43	300	1	29.40
20	-	-	-	0	0	-
21	-	-	-	0	0	-
22	6.69 ± 3.42	5.75 ± 3.42	0.55	3798	13	38.01
23	6.02 ± 3.48	4.91 ± 3.48	0.61	15,407	52	37.06
24	5.78 ± 3.48	4.61 ± 3.48	0.69	14,003	48	37.36
25	5.28 ± 3.37	4.07 ± 3.37	0.68	10,392	35	38.93
26	6.41 ± 3.78	5.17 ± 3.78	0.55	10,537	36	38.72
27	6.19 ± 3.94	4.77 ± 3.94	0.58	4093	14	37.63
28	5.50 ± 3.17	4.49 ± 3.17	0.58	2002	7	42.04
29	6.08 ± 3.41	5.03 ± 3.41	0.65	3832	13	42.92
30	5.61 ± 3.28	4.55 ± 3.28	0.68	1484	5	40.25
31	10.90 ± 4.51	9.93 ± 4.51	0.82	298	1	48.06
32	5.82 ± 3.72	4.48 ± 3.72	0.57	864	3	41.29

**Table 5:** Performance of the neural network model on the test set, aggregated by gEBV. RMSE = root mean squared error; MAE = mean absolute error; Pcorr = Pearson correlation; n. obs = number of observations; n. animals = number of animals in each category. RMSE and MAE are expressed as mean ± standard deviation.

gEBV (kg)	RMSE (kg/day)	MAE (kg/day)	Pcorr	n. Obs	n. Animals	Mean Production (kg/day)
(−296, −96]	4.71 ± 2.67	3.88 ± 2.67	0.62	2048	7	31.01
(−96, 104]	5.01 ± 2.98	4.03 ± 2.98	0.44	4585	16	32.85
(104, 304]	5.43 ± 3.21	4.38 ± 3.21	0.60	6483	22	34.72
(304, 504]	6.07 ± 3.67	4.83 ± 3.67	0.56	4384	15	37.97
(504, 704]	6.76 ± 3.87	5.53 ± 3.87	0.47	8808	30	36.09
(704, 904]	6.10 ± 3.52	4.98 ± 3.52	0.52	8232	28	38.54
(904, 1104]	5.58 ± 3.48	4.36 ± 3.48	0.60	12,089	41	39.46
(1104, 1304]	6.15 ± 3.64	4.96 ± 3.64	0.54	9801	33	41.43
(1304, 1504]	5.90 ± 3.57	4.70 ± 3.57	0.60	4955	17	41.29
(1504, 1704]	6.56 ± 3.53	5.53 ± 3.53	0.57	2658	9	43.81
(1704, 1929)	6.91 ± 4.10	5.57 ± 4.10	0.58	2967	10	42.86

## Data Availability

The original contributions presented in this study are included in the article. Further inquiries can be directed to the corresponding authors.

## Abbreviations

The following abbreviations are used in this manuscript:

FFNN	Feedforward neural network
ML	Machine learning
AMS	Automatic milking system
DIMs	Days in milk
gEBV	Genomic estimated breeding value
RMSE	Root mean squared error
MAE	Mean absolute error
